# Production and validation of model iron-tannate dyed textiles for use as historic textile substitutes in stabilisation treatment studies

**DOI:** 10.1186/1752-153X-6-44

**Published:** 2012-05-22

**Authors:** Helen Wilson, Chris Carr, Marei Hacke

**Affiliations:** 1 Textiles and Paper, The School of Materials, The University of Manchester, Oxford Road, Manchester M13 9PL, UK; 2 Department of Conservation and Scientific Research, British Museum, Great Russell Street, London WC1B 3DG, UK

## Abstract

**Background:**

For millennia, iron-tannate dyes have been used to colour ceremonial and domestic objects shades of black, grey, or brown. Surviving iron-tannate dyed objects are part of our cultural heritage but their existence is threatened by the dye itself which can accelerate oxidation and acid hydrolysis of the substrate. This causes many iron-tannate dyed textiles to discolour and decrease in tensile strength and flexibility at a faster rate than equivalent undyed textiles. The current lack of suitable stabilisation treatments means that many historic iron-tannate dyed objects are rapidly crumbling to dust with the knowledge and value they hold being lost forever.

This paper describes the production, characterisation, and validation of model iron-tannate dyed textiles as substitutes for historic iron-tannate dyed textiles in the development of stabilisation treatments. Spectrophotometry, surface pH, tensile testing, SEM-EDX, and XRF have been used to characterise the model textiles.

**Results:**

On application to textiles, the model dyes imparted mid to dark blue-grey colouration, an immediate tensile strength loss of the textiles and an increase in surface acidity. The dyes introduced significant quantities of iron into the textiles which was distributed in the exterior and interior of the cotton, abaca, and silk fibres but only in the exterior of the wool fibres. As seen with historic iron-tannate dyed objects, the dyed cotton, abaca, and silk textiles lost tensile strength faster and more significantly than undyed equivalents during accelerated thermal ageing and all of the dyed model textiles, most notably the cotton, discoloured more than the undyed equivalents on ageing.

**Conclusions:**

The abaca, cotton, and silk model textiles are judged to be suitable for use as substitutes for cultural heritage materials in the testing of stabilisation treatments.

## Background

**Figure 1:**
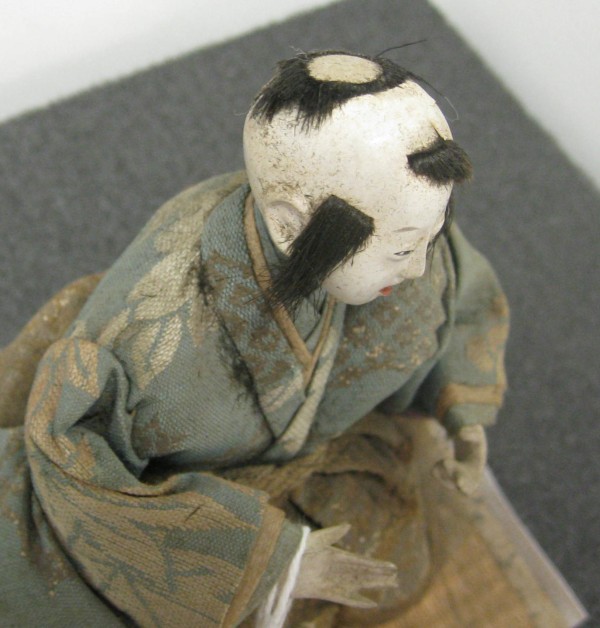
**Losses to the iron-tannate dyed hair (silk) on a Japanese ceremonial Hina doll (British Museum, Department of Asia, AS1981,0808.227). **Image © The Trustees of the British Museum.

**Figure 2:**
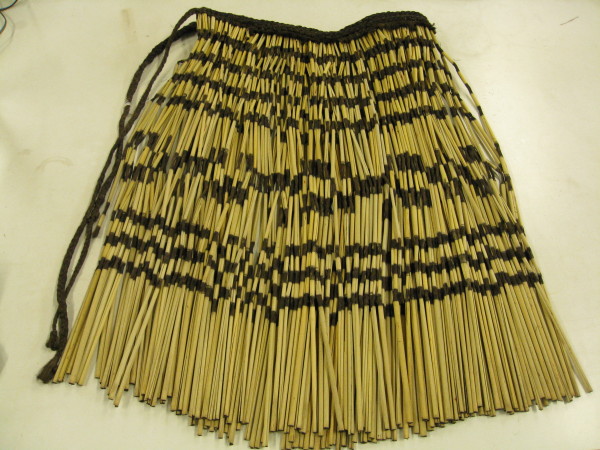
**A Maori *piu piu *(ceremonial skirt), approximately 15 years old, produced from New Zealand flax (*Phormium tenax*) (owned by Dr Vincent Daniels).**

Iron-tannate complexes have been used as inks (iron gall inks) and dyes for thousands of years and are now present in objects of cultural significance worldwide. While iron gall inks have been used predominantly on paper and parchment, iron-tannate dyes have been used to colour a vast array of woven and non-woven materials shades of black, grey, or brown, including proteinaceous materials such as silk (Figure 1), wool, skin, and leather, and cellulosic materials such as cotton, abaca, *Phormium tenax* (New Zealand flax) (Figure 2), and raffia.

Iron-tannate dyes are formed through the combination of iron ions (usually iron(II)) and tannic acids (usually hydrolysable) in water. Historically, iron ions were often sourced from iron-rich mud or iron(II) sulphate (vitriol) while tannic acid (condensed, hydrolysable, or a mixture) was sourced from plant material such as bark, leaves, and galls. Hydrolysable tannins from galls for example include gallotannins and ellagitannins which can be hydrolysed to glucose and gallic acid or ellagic acid, respectively [1]. On combination with ferrous ions hydrolysable tannins form blue-black coloured iron(III)-tannate dye complexes; the colour being due to a reversible charge transfer across the Fe(III)-O bond in the iron(III)-tannate, or iron(III)-gallate, complex [2]. Condensed tannins (proanthocyanidins) are oligomers or polymers of flavan-3-ol (catechin) monomers [1] which form green-black coloured dye complexes on combination with iron(III) ions [3,4]. The exact shade of black, brown, or grey of iron-tannate dyes varies depending on the method of dyeing used and the types and quality of reagents included [5]. Additionally, the dyes can become browner with age as the dye complex is broken down and coloured degradation products such as brown quinones and iron(III) oxides, and yellow ellagic acid are formed [6,7]. See Additional file 1 for further detail on the colour, acidity, and complex structure of iron-tannate dyes.

**Figure 3:**
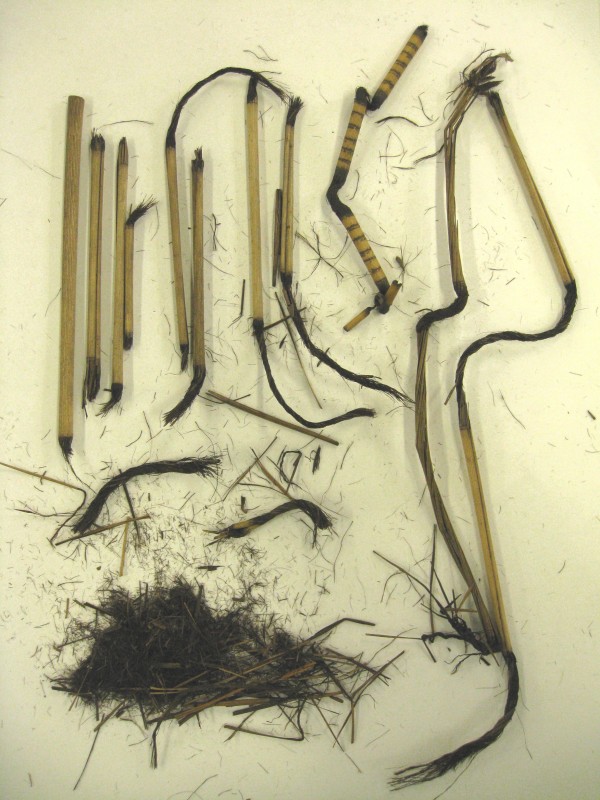
**Remains of a Maori cloak and *piu piu *that has disintegrated in the iron-tannate dyed areas (Horniman Museum).**

Unfortunately, iron gall inks and iron-tannate dyes pose a significant threat to the lifetime of the materials they colour due to their acidity and metal ion content which can accelerate acid hydrolysis and oxidation (see Additional file 1 for more details). This causes tensile strength loss, embrittlement, and discolouration in the substrate. Consequently, many iron-tannate dyed materials are brown rather than black, fragile, exhibit physical losses, or in some cases have crumbled to dust (Figure 3).

While much research has been undertaken into understanding the degradation processes and development of stabilisation treatments for iron gall ink on paper [2,8,9] significantly less research has been undertaken on the iron-tannate dyed textiles that are the focus of this paper [3,4,10-13], and at present there is no suitable non-aqueous treatment with which to stabilise these objects.

In 2008 an AHRC/EPSRC Science and Heritage Programme collaborative PhD project was established at the University of Manchester and the British Museum to investigate non-aqueous stabilisation treatments for iron-tannate dyed organic materials. The use of historic material in these treatment studies was deemed unsuitable for ethical and practical reasons and necessitated the production and use of substitute iron-tannate dyed textiles that:

Exhibit relatively uniform iron and colour distribution to ensure that the iron-catalysed degradation of the dyed textiles occurs as uniformly as possible, thus minimising analytical variability in accelerated ageing and stabilisation treatment studies;

Lose tensile strength and possibly discolour more than undyed equivalent textiles on accelerated ageing, as is seen with naturally aged iron-tannate dyed objects worldwide including in the British Museum’s collection [4,10].

In this paper the production of the substitute textiles is described. The validity for the use of the textiles as substitutes for historic iron-tannate dyed material in accelerated ageing and stabilisation treatment studies is assessed through their characterisation before and after accelerated ageing.

## Production of substitute textiles

Small quantities of iron-tannate dyed silk [12], New Zealand flax [3], and raffia [13] yarns/fibre bundles and textiles have been produced in laboratories by several researchers. For this research significantly larger quantities of uniformly dyed woven textiles were needed and so four textiles (cotton, abaca, silk, and wool) and six specifically developed dye formulations (Table 1) were used on industrial equipment at the University of Manchester’s dyehouse to produce an unprecedented 80 m^2^ of substitute textiles. The pH of the clear and colourless dyebath solutions was tested using pH-Fix 0–14 Fisherbrand pH indicator strips and found to be typically pH 4 to 6 for both tannic acid solutions and metal ion solutions. More detailed information on the development and dyeing of the substitute textiles is presented in Additional file 2.

**Table 1:** Dye formulations used to produce substitute iron-tannate dyed textiles

**Dye code**		**Substrate**	**Liquor: Fabric**^**a**^	**Dyebath A**^**b**^	**Dyebath B**	**Dyebath A+**	**Dyeing sequence**
Proteinaceous	p1	Wool + silk	200 : 3.23	0.02 M FeSO_4_.7H_2_O 55°C, 1 hour	6.5 g.L^-1^ TA 55°C, 3 hours	0.009 M FeSO_4_.7H_2_O 55°C, 1 hour	A B A B A+
	p2	Wool + silk	200 : 3.23	0.02 M FeSO_4_.7H_2_O + 0.002 M CuSO_4_.5H_2_O 55°C, 1 hour	6.5 g.L^-1^ TA 55°C, 3 hours	0.009 M FeSO_4_.7H_2_O + 0.0009 M CuSO_4_.5H_2_O 55°C, 1 hour	A B A B A+
	p3	Silk	200 : 0.62	0.005 M FeSO_4_.7H_2_O 55°C, 1 hour	3.5 g.L^-1^ Gx 55°C, 3 hours	0.002 M FeSO_4_.7H_2_O 55°C, 1 hour	A B A B A+
Cellulosic	c1	Cotton + abaca	60 : 2.01	15 g.L^-1^ TA 20°C, 2 hours	0.04 M FeSO_4_.7H_2_O 20°C, 2 hours	-	A B A B A B
	c2	Cotton + abaca	60 : 2.01	15 g.L^-1^ TA 20°C, 2 hours	0.04 M FeSO_4_.7H_2_O + 0.005 M CuSO_4_.5H_2_O 20°C, 2 hours	-	A B A B A B
	c3	Cotton	60 : 1.22	16.6 g.L^-1^ Gx 20°C, 2 hours	0.024 M FeSO_4_.7H_2_O 20°C, 2 hours	-	A B A B A B

a. Ratio in litres of dyebath : total mass of fabric in kilograms.

b. Abbreviations are as follows: TA: 50:50 mixture of non-purified and purified tannic acid extracts from Chinese galls and sumac, respectively; Gx: non-purified gall powder.

c. In p1, the final quantity of iron (A+) was added directly to the final dyebath B but in p2 and p3, it was applied in a separate dyebath due to substantial and problematic foam formation in p1. Since an unknown quantity of tannic acid remained in the final dyebath B in p1 when the iron (A+) was added, the effective concentration of iron ions available for binding with tannic acid on or within the textile fibres is lower than that in p2 and p3.

## Results and discussion

### Characterisation of unaged iron-tannate dyed model textiles

#### Metal ion content and distribution, including uniformity, in iron-tannate dyed model textiles (XRF and SEM-EDX analysis)

XRF was used to assess the overall metal ion content and uniformity throughout the dyed textiles since an uneven distribution could cause uneven degradation during accelerated ageing.

All iron-tannate dye formulations introduced significant quantities of iron (and copper for the p2 and c2 formulations) into the dyed textiles (Table 2). Dye formulation 3 resulted in the highest levels of iron detected probably due to a greater quantity of tannic acid and gallic acid being present in the aqueous gall powder extract compared to in the mixture of tannic acids used in dye formulations 1 and 2.

**Table 2:** The uniformity of metal ion and colour distribution in unaged substitute textiles determined using XRF and spectrophotometry, respectively

**Sample**	**XRF**^**a**^**(elemental ratios)**		**Colour measurement**		
	**Fe**	**Cu**	L*	a*	b*
Wool undyed (WU)	12 (1)^b^	5 (1)	78.39 (0.39)	−1.06 (0.04)	6.67 (0.24)
Wool dyed with p1 (Wp1)	590 (60)	11 (1)	33.53 (1.03)	1.31 (0.04)	−1.11 (0.12)
Wool dyed with p2 (Wp2)	786 (68)	333 (27)	31.03 (0.79)	0.93 (0.07)	−1.05 (0.18)
Silk undyed (SU)	17 (2)	4 (1)	75.36 (0.36)	−0.20 (0.02)	0.97 (0.12)
Silk dyed with p1 (Sp1)	2124 (526)	9 (5)	20.75 (0.37)	1.46 (0.03)	−3.90 (0.09)
Silk dyed with p2 (Sp2)	2413 (292)	204 (26)	18.76 (0.30)	1.61 (0.03)	−4.37 (0.07)
Silk dyed with p3 (Sp3)	2628 (145)	11 (10)	17.79 (0.20)	1.38 (0.03)	−4.09 (0.07)
Abaca undyed (AU)	24 (5)	4 (3)	74.25 (1.66)	1.91 (0.41)	13.56 (1.09)
Abaca dyed with c1 (Ac1)	1459 (338)	9 (2)	21.66 (0.75)	0.56 (0.05)	−2.22 (0.12)
Abaca dyed with c2 (Ac2)	1490 (190)	371 (53)	23.34 (0.83)	0.50 (0.04)	−2.65 (0.17)
Cotton undyed (CU)	15 (1)	3 (1)	84.31 (0.51)	−0.26 (0.02)	0.67 (0.09)
Cotton dyed with c1 (Cc1)	683 (145)	5 (3)	35.7 (0.91)	0.70 (0.06)	−4.33 (0.15)
Cotton dyed with c2 (Cc2)	742 (41)	83 (11)	33.58 (0.50)	0.80 (0.04)	−4.89 (0.12)
Cotton dyed with c3 (Cc3)	1115 (44)	3 (3)	29.61 (1.01)	0.57 (0.05)	−4.62 (0.13)
Black silk (1881,0802.158 or PRN: RRM 10294^c^)	2370	587	ND^d^	ND	ND
Black dyed North American skin bag (1937,0617.1^c^)	4163	44	ND	ND	ND
Brown braided area of modern *piu piu* owned by Dr Vincent Daniels (Figure 2)	1918	2	ND	ND	ND
Black fibres of broken Maori cloak and *piu piu* from the Horniman Museum (Figure 3)	5924	3	ND	ND	ND

a. Iron and copper content ratios (net elemental peak area: net Compton peak area) multiplied by 1000.

b. Standard deviations of mean data are in parentheses.

c. British Museum registration number.

d. Not done because the samples were too small or the surface too uneven for analysis.

The most uniform metal distributions were achieved with dye formulation 3 (a maximum of 6% variation from the mean) and the least with dye formulation 1 (a maximum of 25% variation from the mean). The production method, particularly the efficacy of the post-dyeing rinsing may have caused these variations in iron content. High levels of iron were also detected in a range of samples from iron-tannate dyed museum objects.

SEM-EDX of the dyed (p1 and c1) and undyed substitute textile cross-sections identified a high variability of iron content in the fibre bundles/yarns, with iron concentration increasing with increasing proximity to the fibre bundle/yarn surface. This variation in the iron content with the location of the fibre within the fibre bundles/yarns will occur throughout the textiles and therefore will not affect the results from tensile testing or colour measurement which will be averaged by analysis of multiple fibres.

**Figure 4:**
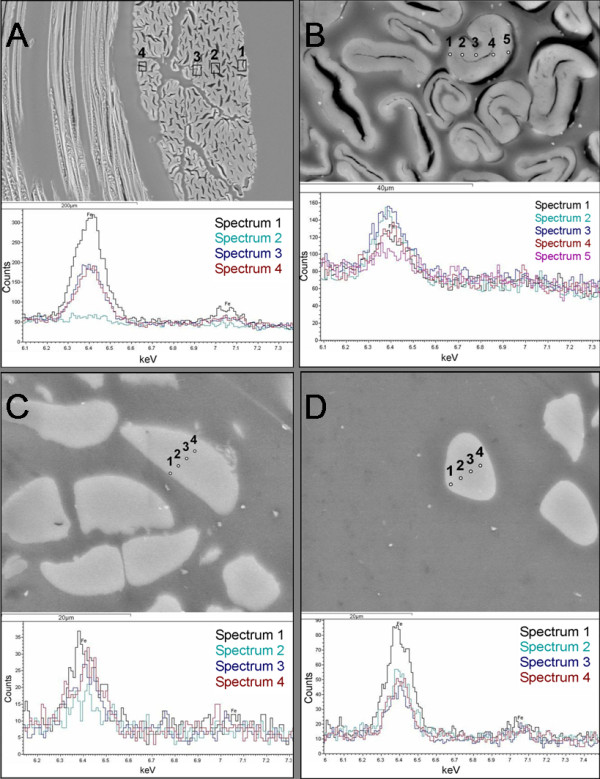
**SEM images and EDX spectra of dyed abaca (A), cotton (B), and silk (C and D) fibres in cross-section.** The dyed silk fibres in C are from the interior of the yarn while those in D are on the crown of the weave.

**Figure 5:**
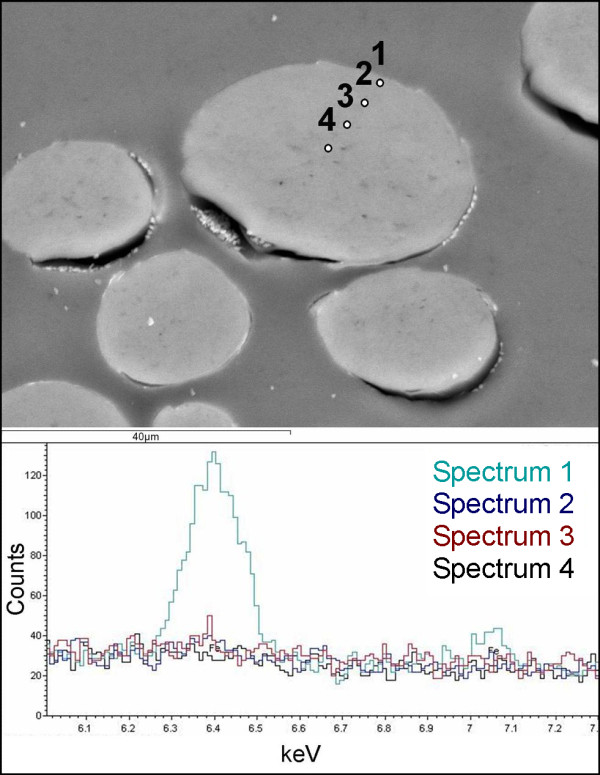
SEM image and EDX spectra of a dyed wool fibre near the crown of the weave.

Importantly, for the individual fibres of abaca, cotton, and silk, iron was easily detected on the exterior and the interior of each fibre and was most concentrated on the exterior (Figure 4). Iron in the wool fibres was primarily located at the exterior of the fibres (cuticle) with minimal or no iron detected inside the fibres (cortex), Figure 5. This is due to the hydrophobic and highly cross-linked cuticle layer present in only the wool fibres, which restricts diffusion of the water-based dye into the cortex of the wool fibres [14]. The lack of a cuticle layer in the silk explains the greater iron content in the silk than in the simultaneously dyed wool (Table 2). Improved dye diffusion into the wool fibres may be achieved through use of a higher temperature such as the 90-100°C usually used for wool dyeing, rather than the 55°C used in these dye formulations. In this study, 55°C was selected in order to minimise thermal damage to the simultaneously dyed silk.

It is likely that the majority of the metal in the substitute textiles is bound in iron-tannate complexes or directly to the fibres since significant or complete removal of water-soluble unbound ions will have occurred in the post-dyeing rinsing. The iron-tannate dye complexes can be physically bonded to the textile fibres via Van der Waals’ forces [15-17] or chemically bonded via the mordant of the dye. In the proteinaceous dye formulations, the metal ions acted as mordants so that fibre/iron/tannic acid interactions will predominate [18]; in the cellulosic dye formulations tannic acid was the mordant and so fibre/tannic acid/iron interactions will predominate. Iron ions and copper ions can bind to hydroxyl, carbonyl, and carboxyl groups in proteinaceous and cellulosic textiles as well as to amine, amide, and thiol groups present in proteinaceous textiles [15,16,19]. Copper ions bind more strongly than iron ions, particularly to thiols [20-22]. Carboxylate anion groups are the major binding sites in wool [20] and silk [23]. The isoelectric points of wool and silk are approximately at pH 5.6 and 2.8, respectively [24], and are the pH values at which the proteins are electrically neutral, having equal quantities of positive (e.g. –NH_3_^+^) and negative (e.g. –COO^-^) functional groups. Since the pH of the dyebaths for the model textiles ranged between pH 4 and 6, it is likely that the silk fibroin will be slightly negatively charged which will attract the metal cations, while the wool will be either slightly positively charged which will repel the metal cations, or will be electrically neutral. In the silk the metal ions can bind by co-ordinate bonds to un-ionised groups such as amines and hydroxyl groups as well as by ionic bonds to negatively charged groups such as carboxylate and sulphonate groups [25-27]. In the wool the metal ions will be repelled by positively charged groups such as protonated amines but can bind to un-ionised groups such as amine groups, and to the ionised carboxyl groups that account for the majority or all of the carboxyl groups present in the wool since the pH of dye baths are close to the isoelectric point of wool. Wool p2 contains more copper ions than silk p2 because of its greater aspartic acid, glutamic acid [28], and thiol content [15].

The carbonyl, carboxyl, and hydroxyl groups in cellulosic materials can bind to tannic acid (by hydrogen bonding) as well as to metal ions [15,16]. Dyed abaca contains more iron and copper than equivalently dyed cotton probably because of the greater presence of non-cellulosic components such as lignin and hemicellulose which also contain hydroxyl and carboxyl groups [29,30].

#### Iron-tannate dyed textile colour and colour uniformity

**Figure 6:**
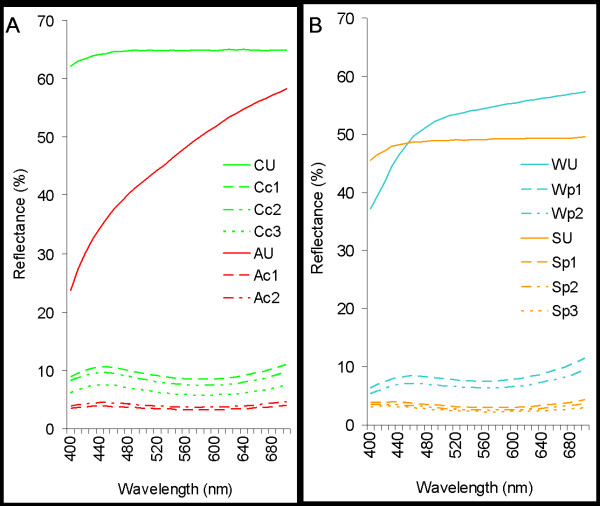
Visible reflectance spectra of the unaged cellulosic (A) and proteinaceous (B) substitute textiles.

The colour of textiles can be described using reflectance spectra such as those in Figure 6, or quantified using co-ordinates of a colour space system such as CIE L* a* b* (Table 2). The co-ordinate values L*, a*, and b* correspond to the blackness (L* = 0), whiteness (L* = 100), redness (+a), greenness (−a), yellowness (+b), and blueness (−b), respectively [31]. A uniform colour distribution is needed to minimise error in characterising colour changes associated with ageing and stabilisation treatment studies.

All of the iron-tannate dyes caused similar mid to dark blue/grey colouration of the substitute textiles (Figure 6). The colour is due to a charge transfer in the iron-tannate dye complex [2] which causes a relatively strong absorption of red light (600–700 nm with an absorption maximum at pH 4 of 620 nm [6]). Comparable reflectance spectra have been reported with laboratory produced iron gall ink [6] and traditionally dyed *Phormium tenax* (New Zealand flax) [32]. Increasing levels of iron in the textiles (Table 2) correlate well with their L*, a*, and b* values.

Examination of the dyed fabrics indicated that relatively uniform textile colouration was achieved with variations in L*, a*, and b* being generally less than 10% of the mean.

#### Surface pH of model textiles

Iron-tannate dyed textiles are typically acidic, primarily due to the hydroxyl and carboxyl functionalities of the tannic acid (see Additional file 1 for more details). This is demonstrated by the surface pH of iron-tannate dyed museum objects (Table 3) and the aqueous pH results from the same or similar iron-tannate dyed objects reported in the literature [10,33]. Correspondingly, the dyed substitute textiles were found to be acidic, exhibiting surface pH values between 2.65 and 3.91 which is significantly lower than the surface pH of the undyed equivalents which ranged between pH 5.36 and 7.46 (Table 3).

**Table 3:** The surface pH, breaking load, and extension of the unaged substitute textiles and iron-tannate dyed museum objects

**Sample**	**Surface pH**	**Tensile testing**	
		**Breaking load (N)**	**Extension (%)**
Wool undyed (WU)	7.46 (0.49)^a^	ND^b^	ND^b^
Wool dyed with p1 (Wp1)	3.91 (0.10)	ND	ND
Wool dyed with p2 (Wp2)	3.84 (0.11)	ND	ND
Silk undyed (SU)	7.24 (0.09)	70.2 (4.9)	28.2 (1.7)
Silk dyed with p1 (Sp1)	3.60 (0.06)	62.9 (4.9)	25.0 (2.6)
Silk dyed with p2 (Sp2)	3.69 (0.09)	56.4 (2.5)	22.9 (1.7)
Silk dyed with p3 (Sp3)	3.57 (0.06)	55.3 (3.8)	22.6 (1.6)
Abaca undyed (AU)	5.36 (0.18)	239.9 (43.1)	3.9 (0.5)
Abaca dyed with c1 (Ac1)	2.86 (0.07)	105.9 (18.3)	2.1 (0.3)
Abaca dyed with c2 (Ac2)	2.67 (0.08)	130.1 (24.8)	2.5 (0.5)
Cotton undyed (CU)	6.61 (0.11)	73.2 (7.7)	10.5 (1.1)
Cotton dyed with c1 (Cc1)	2.72 (0.06)	68.3 (5.3)	6.9 (0.7)
Cotton dyed with c2 (Cc2)	2.65 (0.07)	51.0 (4.8)	9.2 (1.3)
Cotton dyed with c3 (Cc3)	2.48 (0.04)	45.7 (8.6)	10.2 (1.9)
Dyed areas of modern *piu piu* owned by Dr Vincent Daniels (Figure 2)	3.72 (0.28)	ND	ND
Black fibres of broken Maori cloak and *piu piu* from the Horniman Museum (Figure 3)	2.89 (0.19)	ND	ND
Black cotton Akali Sikh turban (2005,7-27.1^c^)	3.39	ND	ND

a. Standard deviations are noted in parentheses when more than one analysis of a sample was taken.

b. Not done due to either slippage of sample in the jaws of the tensile tester (wool samples) or the samples being too small to test (historic samples).

c. British Museum registration number.

#### Changes in substitute textile tensile strength and extensibility due to dye application

Generally, the application of the dyes caused significant loss of tensile strength (breaking load) and extensibility in the textiles, even before any accelerated ageing had occurred (Table 3). Dyeing of abaca caused the greatest tensile strength loss of all the substitute textiles, followed by cotton, and finally silk. Wool was not tested since the high tensile strength of the wool led to unacceptable slippage of the sample during testing. The damage could be due to the acidity (pH 4 to 6) and, for the silk, the elevated temperature (55°C) of the dyebath solutions. Harsh dyeing conditions could be a major factor in the tensile strength loss seen in historic iron-tannate dyed textiles, especially as soluble iron ions and acid can be removed from the textiles during post-dyeing rinsing [34].

### Characterisation of model textiles following accelerated ageing (tensile testing and spectrophotometry)

Despite showing the greatest variation in iron ion distribution (Table 2), the c1 and p1 substitute textiles were chosen to be aged as they were dyed with the highest purity and most essential reagents (iron ions and tannic acids) only, thus minimising the influence of impurities.

**Figure 7:**
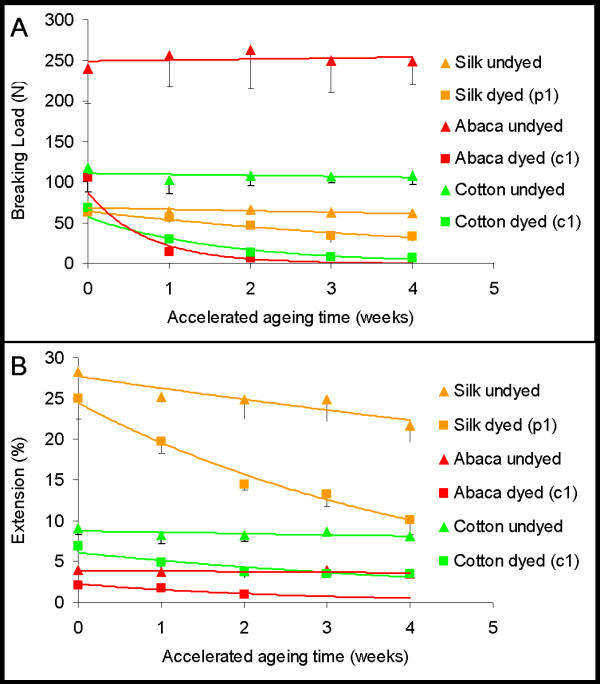
Effect of accelerated ageing on the breaking load (A) and extension (B) of the substitute textiles.

Little or no change in tensile strength (breaking load) or extensibility was seen in the undyed materials after four weeks of ageing. However, significant loss of tensile strength and extensibility occurred in the dyed abaca and cotton (Ac1 and Cc1) after one week of accelerated ageing and in the dyed silk (Sp1) after two weeks of ageing (Figure 7 and Table 4). The extent of degradation exhibited by the dyed textiles correlates well with their initial surface pH (Table 3), iron content (Table 2), and the presence of iron in the structurally important internal areas of the dyed fibres, suggesting that the degradation has occurred by acid hydrolysis and metal-catalysed oxidation, similar to that observed in historic iron-tannate dyed textiles [10]. The proportion of degradation occurring by the two mechanisms may be different to those experienced during natural ageing due to the elevated environmental conditions during accelerated ageing, but the essential result of catalysed loss of tensile strength and extensibility of iron-tannate dyed textiles has been determined.

**Table 4:** Changes in colour, tensile breaking load (N), and extension (%) of substitute textiles during accelerated ageing (80°C, 58% RH)

**Sample**	**Extent of ageing (weeks)**	**Difference in colour (aged versus unaged)**				**Mean tensile properties**	
		**ΔE**_00_*	ΔL*	Δa*	Δb*	**Breaking load (N)**	**Extension (%)**
WU	0	0.00	0.00	0.00	0.00	ND^a^	ND
	1	1.14	0.05	−0.40	1.38	ND	ND
	2	1.33	0.32	−0.48	1.58	ND	ND
	3	1.75	0.17	−0.61	2.16	ND	ND
	4	2.44	−0.22	−0.72	3.17	ND	ND
SU	0	0.00	0.00	0.00	0.00	70.2 (4.9)^b^	28.2 (1.7)^b^
	1	1.04	−0.06	−0.32	1.00	64.2 (4.4)	25.2 (0.8)
	2	1.57	0.00	−0.44	1.54	65.5 (4.8)	24.9 (2.5)
	3	2.16	−0.48	−0.54	2.17	62.3 (4.7)	24.8 (2.7)
	4	2.59	−0.40	−0.57	2.70	61.6 (4.3)	21.7 (2.1)
AU	0	0.00	0.00	0.00	0.00	239.9 (43.1)	3.9 (0.5)
	1	2.39	−2.17	0.67	2.78	255.5 (38.1)	3.7 (0.5)
	2	3.08	−2.82	1.05	3.43	262.8 (47.3)	3.8 (0.5)
	3	3.03	−1.63	1.39	4.22	250.0 (40.3)	3.9 (0.6)
	4	3.95	−2.64	1.76	5.29	248.8 (28.9)	3.5 (0.5)
CU	0	0.00	0.00	0.00	0.00	117.1 (9.2)	9.1 (0.8)
	1	0.62	−0.15	−0.10	0.61	101.9 (15.7)	8.2 (1.0)
	2	1.20	0.06	−0.06	1.26	107.2 (11.6)	8.2 (0.7)
	3	1.52	−0.84	−0.07	1.48	107.1 (8.3)	8.6 (0.5)
	4	1.82	−0.28	−0.06	1.92	108.2 (11.0)	8.1 (0.5)
Wp1	0	0.00	0.00	0.00	0.00	ND	ND
	1	ND	ND	ND	ND	ND	ND
	2	2.74	−0.50	−0.39	2.73	ND	ND
	3	3.64	0.42	−0.46	3.70	ND	ND
	4	5.70	3.33	−0.62	5.21	ND	ND
Sp1	0	0.00	0.00	0.00	0.00	62.9 (4.9)	25.0 (2.6)
	1	1.07	−0.61	−0.17	1.13	55.6 (6.1)	19.7 (1.4)
	2	1.78	−0.31	−0.30	1.98	46.0 (3.7)	14.5 (0.7)
	3	2.15	0.25	−0.41	2.37	34.2 (9.4)	13.2 (1.5)
	4	3.27	2.02	−0.39	3.24	33.2 (6.1)	10.1 (1.8)
Ac1	0	0.00	0.00	0.00	0.00	105.9 (18.3)	2.1 (0.3)
	1	3.04	0.24	0.11	3.17	14.6 (4.9)	1.8 (0.4)
	2	4.54	−0.37	0.63	4.64	6.5 (1.9)	1.0 (0.3)
	3	5.33	−0.68	1.20	5.34	ND^c^	ND^c^
	4	4.96	−2.00	1.08	4.75	ND^c^	ND^c^
Cc1	0	0.00	0.00	0.00	0.00	68.3 (5.3)	6.9 (0.7)
	1	7.35	−4.23	0.30	6.92	29.5 (3.9)	4.9 (0.6)
	2	10.49	−4.68	1.29	10.55	13.0 (0.4)	3.7 (0.6)
	3	12.15	−5.71	2.29	12.24	7.7 (1.1)	3.5 (0.2)
	4	13.24	−6.72	3.02	13.22	6.2 (0.7)	3.5 (0.2)

a. Not done.

b. Standard deviations of mean data are in parentheses.

c. Not done because the samples were too brittle to be prepared for analysis.

Ac1 lost tensile strength and extensibility faster than Cc1 and was too fragile for tensile testing after 2 weeks of ageing. This faster rate of degradation is congruent with the greater presence of non-cellulosic components such as hemicellulose [29,30], and the higher iron content (Table 2) in Ac1 than Cc1.

The L*, a*, b* colour coordinates of a sample identify a point in 3D CIELAB colour space that describes the colour of the sample. The colour difference between two samples, e.g. between the aged and unaged substitute textiles, is described by ΔE_00_* which is the distance in 3D CIELAB colour space between the points that describe the colour of these samples. The CIE2000 colour difference formula that is based on the law of Pythagoras is used to calculate ΔE_00_* [31,35]. Depending on various factors such as surface texture, background, and viewing angle, 50% of observers can perceive a colour difference between samples of ΔE_00_* = 1, while the majority can perceive a colour difference of 3 or more [36].

**Figure 8:**
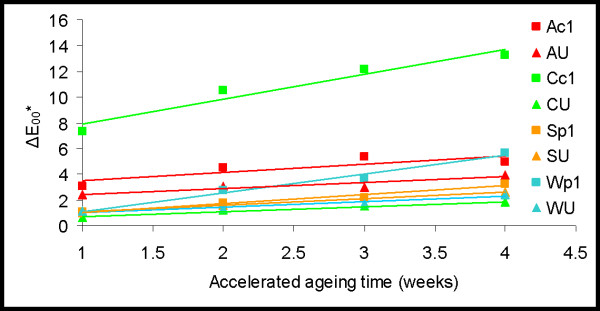
**The effect of accelerated ageing on the colour difference (ΔE**_00_*) of substitute textiles.

After 4 weeks of accelerated ageing there was little overall change in colour of the undyed textiles (ΔE_00_* < 4) (Figure 8 and Table 4). The dyed textiles (p1 and c1) changed colour more than the simultaneously aged undyed equivalents. The dyed cotton showed significantly greater colour change (ΔE_00_* = 13.24) than the other dyed textiles (ΔE_00_* < 6).

**Figure 9:**
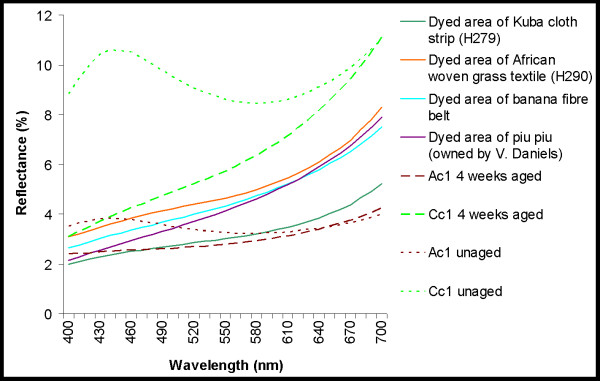
Visible reflectance spectra of cellulosic substitute textiles after 0 and 4 weeks of accelerated ageing (80°C, 58% RH) and of cellulosic museum objects.

More specifically, a small yellowing (+Δb*) of the undyed textiles occurred during accelerated ageing which for the cotton, wool, and silk was less than that seen in the dyed equivalents. The dyed textiles (Figure 9 and Table 4) showed an increase in redness (+Δa* and a greater reflectance of 600–700 nm light), particularly for the dyed cotton and abaca, and yellowness (+Δb* and a greater reflectance of 560–600 nm light) with age. These results are explained by the breakdown of the blue-black iron-tannate dye complex with thermal ageing as previously described [6,7] (see also Additional file 1), which has been observed with model iron gall inks on paper and traditionally dyed New Zealand flax on ageing [6,32]. The reflectance spectra of the four week accelerated aged dyed cotton and abaca correlate well with the reflectance spectra of the cellulosic museum objects analysed (Figure 9).

## Experimental

### Dyeings

The dyeings were performed on a Winch and a Jigger machine at the University of Manchester’s dyehouse. Further information including material sources can be found in Additional file 2.

### Accelerated ageing

Substitute textiles were accelerated aged in two stacks (one for the dyed and one for the undyed samples) at 80°C and 58% RH for 1, 2, 3, and 4 weeks in a Sanyo Gallenkamp Environmental Chamber. These are similar to the conditions used in iron gall ink studies (80°C, 65% RH) [37]. The stacks were arranged in the order of abaca, cotton, silk, and wool from the shelf upwards. The sample stacks were not rotated during ageing but were moved around on the shelf throughout ageing to counter any location-dependant variations in temperature and relative humidity in the chamber. See Additional file 3: Experimental section for more details.

### Analytical techniques

Characterisation of the unaged substitute textiles and historic material was achieved using XRF, spectrophotometry, tensile testing, SEM-EDX, and surface pH testing. The aged substitute textiles were characterised using spectrophotometry and tensile testing. Brief methodologies for these techniques are described below. See Additional file 3: Experimental section for further details.

#### XRF

A Bruker ArtTax μ-XRF spectrometer with a molybdenum X-ray tube and ArtTax4.9 software was used to analyse the unaged substitute textiles and the historic samples semi-quantitatively. Single thicknesses of substitute textiles were analysed in 8 locations on filter paper for 100 s, using a 1.5 mm collimator, 50 kV, and 500 μA in air. Analysis of material from museum objects occurred with and without helium purging over 100-400 s using a 0.65 mm or 1.5 mm collimator, 50 kV, and 500 μA.

Elemental peak areas were divided by the Compton peak area and multiplied by 1000 to give the XRF ratio values that are reported in Table 2. By normalising the elemental peak areas to the Compton peak area the XRF ratios acquired using different analytical methods are comparable.

#### SEM-EDX

Resin mounted cross-sections of dyed (p1 and c1) and undyed substitute textile fabrics were analysed using an Hitachi S-4800 Field Emission SEM and an Hitachi variable pressure S-3700 N SEM (operating at 30 Pa). The SEMs were operated at 20 kV and a 12 mm working distance for all analyses. Analysis was conducted using Oxford Instruments energy dispersive X-ray analysers with INCA software. EDX spectra were collected for varying livetimes after optimisation of the iron peak versus total time taken for analysis: 200 s for abaca and silk; 200–300 s for cotton and 500–1000 s for wool. Dyed and undyed samples of the same material were analysed using the same conditions for comparison.

#### Surface pH analysis

Individual sheets of substitute textile were laid on a clean glass sheet and a drop of deionised water added. A Mettler Toledo InLab®Surface pH electrode attached to a Hanna Instruments HI2210 pH meter with temperature probe was then applied to the wetted area and held in place until the pH value stabilised. Ten analyses per substitute textile were made on randomly selected locations of randomly selected textile sheets. pH 4.01 and pH 7.01 buffer solutions were used to calibrate the equipment prior to analysis.

Samples of museum objects were analysed as above one and four times depending on sample size.

#### Tensile testing

70 – 100 mm long strips of cotton and silk textiles (10 mm wide) and strips of abaca textiles (11 fibre bundles wide) were tested using an Instron 4411 tensile tester with 500 N static load cell and Series IX software. The warp direction of the cotton, abaca and silk fabrics was tested. The strips had been conditioned to approximately 21°C and 50% RH overnight before testing. Between eight and ten strips were analysed per sample (as sample size allowed) using a 50 mm gauge length and 10 mm min^-1^ extension speed as used by Garside, Wyeth and Zhang [38]. Exponential trend lines were fitted to tensile testing data using MS Excel.

#### Colour measurement

Average L*, a*, b* values of SCI/100 and SCE/100 data were collected using a Konica/Minolta CM-2600d spectrophotometer, Spectramagic 3.60 software and the following settings: SCI + SCE, medium aperture, UV included, 10° observer and D65 illuminant. The spectrophotometer was calibrated using a white standard before analysis and the textiles were analysed on black velvet.

10 randomly selected sheets of each unaged substitute textile were analysed in 3 randomly selected locations while each aged substitute textile sample was analysed in 5 randomly selected locations. Single layers of textile were analysed except for the unaged abaca textiles which were folded so that two layers were measured simultaneously due to the looseness of the weave compared to the other textiles. Aged abaca was too brittle to be folded without breaking and so one layer of aged abaca was measured at a time.

CIE2000 was used to calculate the ΔE_00_*, ΔL*, Δa*, Δb* from SCE/100 data from the aged textile compared to the unaged equivalent textile.

The small aperture rather than medium aperture was used to analyse up to three areas of the historic samples as sample size allowed. All other conditions were the same as for spectrophotometry of substitute textiles.

## Conclusions

Cotton, abaca, wool, and silk iron-tannate dyed substitute fabrics have been produced on a large and unprecedented scale for use in stabilisation treatment studies. The achieved colours were characteristic of iron-tannate complexes. The harsh dyeing conditions led to immediate deterioration of mechanical properties of the textiles. Dyeing introduced significant acid and metal ion content to the textiles which was shown to be present in the structurally important internal areas of the dyed cotton, abaca, and silk fibres. The use of a higher temperature during dyeing would have improved dye diffusion into the internal areas of the wool fibres. Colour, surface pH, and metal ion content were found to be suitably uniform across the textiles for the needs of this accelerated ageing study and future stabilisation treatment studies.

The dyed cotton, abaca, and silk substitute textiles lost tensile strength and extensibility significantly faster than undyed equivalents on accelerated thermal ageing, as has been known for hundreds of years to occur to iron-tannate dyed objects. Discolouration of the dyed textiles was also observed during accelerated ageing due to the breakdown of the iron-tannate dye complex resulting in colours of cellulosic textiles being comparable to the colours of naturally aged cellulosic museum objects. Consequently, the cotton, abaca, and silk model textiles were found to be valid substitutes for historic iron-tannate dyed textiles in stabilisation treatment studies.

## Abbreviations

SEM-EDX: Scanning Electron Microscopy-Energy Dispersive X-ray Microanalysis; XRF: X-ray Fluorescence; C: Cotton; A: Abaca (*Musa textilis*); W: Wool; S: Silk; p1-3: Dye formulations 1–3 described in Table 1 for proteinaceous textiles; c1-3: Dye formulations 1–3 described in Table 1 for cellulosic textiles; U: Undyed model textile; CIE2000: Commission Internationale de L’Eclairage 2000 colour space formula.

## Competing interests

The authors declare that they have no competing interests.

## Authors’ contributions

MH and CC proposed the project. HW developed the dye formulations used on the model textiles and with assistance from Phil Cohen from the University of Manchester, produced the dyed model textiles in the dyehouse at the University of Manchester. HW performed all the analyses and data interpretation presented. Supervision was provided when required by CC and MH. All authors read and approved the final manuscript.

## Supplementary Material

Additional file 1 **Iron-tannate dye chemistry.** Text and schemes [2-7,9,10,39-61]. Click here for file

Additional file 2 **Further substitute textile development and dyeing method details.** Text and images [62-66]. Click here for file

Additional file 3 **Experimental section.** Text only [37,38,67]. Click here for file
