# Supplementary material for: Production and validation of model iron-tannate dyed textiles for use as historic textile substitutes in stabilisation treatment studies
Source: Chem Cent J. 2012 May 22;6:44. doi: 10.1186/1752-153X-6-44 (PMC3495704; doi:10.1186/1752-153X-6-44)
Supplement: Additional file 1 — Iron-tannate dye chemistry. Text and schemes [2-7,9,10,39-61]. [file 1752-153X-6-44-S1.doc]

Additional file 1: Iron-tannate dye chemistry

Iron-tannate dyes are formed by combining iron ions (frequently ferrous ions), and tannic acids (frequently hydrolysable) in water. Details of sources and definitions of tannin types are noted in the Background section of the paper.

**1. Iron-tannate dye structure**

The nature of the complexes present in iron-tannate dyes is still not fully understood. Several studies have been undertaken in which iron(II) ions, iron(III) ions, or metallic iron have been combined with hydrolysable tannic acids or its phenolic hydrolysis products such as gallic acid, ellagic acid, and pyrogallol [2, 39–45]. These have demonstrated the pH dependence of the stability of the complexes. Under acidic conditions (as occurs in iron-tannate dyes) 1:1, 1:2, and 2:2 (Scheme 1) iron(III):gallic acid complexes [2, 43, 45–47] and 1:1, 1:2, and 2:1 iron(III):tannic acid complexes [44, 48] have been identified. Under neutral and alkaline conditions 1:2 and 1:3 iron(III):gallic acid complexes [49] and 2:1 and 4:1 iron(III):tannic acid complexes [44] have been identified. Iron(II) complexes such as that in Scheme 1 have also been evidenced [45] and are possible despite the rapid oxidation of iron(II) to iron(III) in air, due to direct combination of iron(II) with polyphenols and reduction of bound and unbound iron(III) by polyphenols [45, 49]. Indeed, approximately 15% to 52% of total iron content in iron-gall inks on a range of historic documents and model samples was iron(II) [50, 51, 9]. A large hexagonal 1:1 iron(III):gallic acid framework has also been proposed in which each iron(III) ion is bound to four gallic acid molecules in octahedral arrangement [39]. However, this was formed by combining iron(III) chloride with gallic acid on a gel matrix rather than combining in solution as occurs during the production of iron-gall inks and iron-tannate dyes. Consequently, it is uncertain if this framework is present in the either the inks or dyes.

Scheme 1. The formation of the blue-black iron(III)-gallic acid complexes (c) and (d) as proposed by Krekel [2]. 3,4,5-trihydroxybenzoic acid (gallic acid) (a) combines with aqueous iron(II) ions to produce a water-soluble, colourless 1:1 iron(II)-gallate complex (b) and acid. Oxidation of (b) produces an insoluble blue-black 2:2 iron(III)-gallate complex (c). Decarboxylation of (c) catalysed by unbound iron(III) ions results in the iron(III) pyrogallol complex (d).

**2. Iron-tannate dye colour**

Iron-tannate dyed organic materials are often black but can also be grey or brown immediately after dyeing and often turn browner with age. Factors affecting the colour of the dyed material include:

a. The iron-tannate dye complex

The blue-black colour of complexes of hydrolysable tannins and their hydrolysis products and iron(III) is due to a reversible light-induced charge-transfer across an Fe-O bond of the complex (Scheme 2) [2]. The combination of condensed tannins and iron(III) ions produces green-black complexes [3, 4].

Scheme 2. Charge-transfer in an iron(III) pyrogallol complex [2]

b. Iron-tannate dye formulation

The reagents used including their purity, source, quantity, and freshness affect the colour of the dyed material. For tannic acid sources the time of year when the source was harvested and length of time between harvesting and use is important as the quantity of tannic acid in a source varies throughout the year and decreases over time once harvested. The production method including length of exposure to the reagents, pH of dyeing solutions, temperature of solutions, and the water supply can also affect the colour achieved by the dye [5].

c. Coloured degradation products

Oxidation and acid hydrolysis of the substrate, breakdown of the iron-tannate dye complex, and oxidation of polyphenols can all lead to coloured degradation products [52, 6].

Acid catalysed breakdown of the dye complex through interaction with light forms 1,2-benzoquinone (Scheme 3). This then forms brown degradation compounds such as ellagic acid (yellow-brown) (**f**) and purpurogallin (reddish brown) (**g**). Gallic acid and ellagic acid can be further degraded to produce polymeric compounds similar to humic acid [53, 7]. The dyebaths for the model textiles developed for this research were around pH 5 and the surface pH of the resulting dyed textiles range from pH 2-4 (See Table 3 in the paper).

Scheme 3. Acid-catalysed degradation of iron(III) pyrogallol [6]

Above pH 8 the black complex in iron gall ink is dissolved as phenols are irreversibly oxidised. A reddish brown solution including purpurogallin is formed [2].

The presence of high quantities of iron(III) in the dye will increase the brown tone of the dye since the iron(III) will oxidise the gallic acid to form brown oxidation products and iron(III) oxides can form [2, 7].

**3. Iron-tannate dye acidity**

Iron-tannate dyes and iron gall inks are highly acidic (the pH of fresh iron gall inks has been found to range from 1.5 to 3.7 [2, 54]) due to:

1. the iron source - Unbound iron contributes to the acidity by forming acidic hydration products [55, 56]. The counter ion of the iron can form acidic compounds during iron-tannate or iron-gallate complex formation such as sulphuric acid [2];
2. the tannin source - The tannic or gallic acids contain labile hydroxyl and carboxyl groups which can contribute to the acidity when uncomplexed with iron; the pH of dissolved tannins varies for tannin type but for tannic acid (10 g/L) is 2.8 [57];
3. other reagents - The inclusion of other reagents will affect the acidity, for example vinegar will lower the pH while urine will raise it.

When applied to a substrate the pH of the ink or dye can be altered by components of the substrate such as calcium carbonate in parchment, and alum in paper. Analysis of historic iron gall ink documents has identified ink lines of pH 3.7 - 7.1 [58] and the aqueous pH of an Akali Sikh turban (British Museum registration number 2005,7-27.1) was 4.1 [33]. Unlike for iron-gall ink on documents, iron-tannate dyed textiles are often washed after dyeing. This can remove water-soluble acidic components from the textile such as unbound iron ions and tannic acid, and sulphuric acid for example. Consequently, in washed iron-tannate dyed textiles the majority of the high acidity will be due to bound tannic acid. The acidity of the dyed or inked substrate can also increase due to oxidation of the substrate. Modern iron-tannate dyed New Zealand Flax from a *piu piu* (ceremonial skirt produced by the Maori) was found to have a pH of 4.5 – 6.5 while an historic equivalent had a pH of 3.5 – 4.3 [10].

**4. Catalytic nature of iron-tannate dyes**

The damaging effect of iron-tannate dyes on the substrate has been known for centuries and is due to acid catalysed hydrolysis and metal ion catalysed oxidation [4]. The acidity of the dyed substrate due to the dye and oxidation of the substrate accelerates the rate of acid hydrolysis which fragments the polymer chains, decreasing the mechanical strength of the fibres.

Iron ions present in the dye complex are not catalytically active unless the dye complex is broken down; then in addition to any unbound iron ions originating from an excess of iron in the dye formulation, the ions can catalyse oxidation either directly or via the Fenton reaction [59]. This causes the formation of cross-links between polymer chains, introduces acid groups along the chains, and fragments the polymer chains thus embrittling, acidifying, and weakening the substrate. In cellulosic fibres iron(III) ions bound to carboxylic acid groups of hemicellulose can cause light or heat initiated decarboxylation which generates free radicals which can also fragment the cellulose [3]. Other unbound metal ions such as copper can also accelerate oxidation (sometimes more effectively than iron [60]), however these are likely to be present in significantly lower concentration than the iron ions [61]. Via the Fenton reaction iron(III) ions are converted back to iron(II) ions enabling oxidation to continue. Consequently, many iron-tannate dyed materials are fragile and exhibit physical losses. In some cases iron-tannate dyed objects have crumbled to dust (Figure 3) and at present there is no suitable non-aqueous treatment with which to stabilise these objects.
