# Supplementary material for: Production and validation of model iron-tannate dyed textiles for use as historic textile substitutes in stabilisation treatment studies
Source: Chem Cent J. 2012 May 22;6:44. doi: 10.1186/1752-153X-6-44 (PMC3495704; doi:10.1186/1752-153X-6-44)
Supplement: Additional file 2 — Further substitute textile development and dyeing method details. Text and images [62-66]. [file 1752-153X-6-44-S2.doc]

Additional file 2: Further substitute textile development and dyeing method details

**1. Further information on development of the substitute dyed textiles**

Many of the iron-tannate dyed organic materials in the British Museum’s collections are cellulosic (including cotton, raffia, New Zealand flax, and abaca) with a significantly smaller quantity being proteinaceous (wool, silk, and leather). This was ascertained following discussion with British Museum curators and conservators regarding black dyed objects of poor physical condition, visual inspection of black dyed organic materials within the British Museum’s collections, and investigation using the British Museum’s object records database.

Woven textiles rather than individual fibres were the chosen textile form because of the increased sample size this would allow for characterisation. Larger sample sizes increase the sample uniformity, improve handle-ability and enable aesthetic changes due to treatments to become noticeable. Importantly, woven textiles are closer to the form of many of the iron-tannate dyed British Museum objects that need stabilising.

Critical evaluation of primarily Western historic iron-tannate dye recipes from the 16^th^ to 20^th^ centuries [62–66] identified a range of variables that were investigated in small scale laboratory experiments. These included variations in temperatures, immersion times, number and type of repeat dyeings, and method of application of reagents i.e. application of iron and tannin sources simultaneously via one solution, or the separate application of each reagent, and the order of application in the latter case. Reagent types, concentrations, and combinations (such as inclusion or exclusion of copper sulphate and use of purified tannic acids or aqueous extract of gall powder) were also investigated.

Dye formulations derived from the small scale experiments were further optimised at the University of Manchester’s dyehouse where 5-15 m^2^ of 14 substitute textiles were produced.

**2. Further information on textile dyeing method**

The final formulations differed for proteinaceous and cellulosic materials (p1-3 and c1-3, respectively) and are summarised in Table 1. The fabrics were pre-washed using sodium carbonate and non-ionic detergent. Due to practicalities of handling, wool and silk were dyed together in a Winch machine (Figures 1 and 2) and cotton and abaca were dyed together in a Dye Jigger machine (Figures 3 and 4). The dyebaths were made up fresh for each application. The reagents were dissolved in a small amount of water before adding them to the dyebaths and diluting to the correct volume. The non-purified tannic acid extract from Chinese galls and the purified tannic acid extract from sumac were fully soluble in water and were used as received. However, the non-purified gall powder was only partially soluble and therefore, the gall powder was extracted by soaking in a bucket of tap water for several days prior to dyeing. The concentrated extract was then diluted to the correct volume in the dyebaths. Once the last tannic acid bath had been completed for wool and silk (B), additional metal ions (iron sulphate and copper sulphate) were added to the bath (A+) which was then left for 1h before washing as usual. The fabrics were rinsed twice in tap water for five minutes between dyebaths and after the final dyebath. All fabrics except abaca which was air dried due to its stiffness, were dried using a hydroextractor and tumble drier with low mechanical action.

**3. Material sources**

VWR (https://www.vwrsp.com/index.cgi) supplied the iron(II) sulphate heptahydrate (CAS: 7782-63-1) and copper(II) sulphate pentahydrate (CAS: 7758-99-9) used in the laboratory experiments. Sigma Aldrich supplied the iron(II) sulphate heptahydrate (7782-63-0) and copper(II) sulphate pentahydrate, 98% (7758-99-8) used at the University of Manchester’s dyehouse. Ajinomoto OmniChem (http://www.natural-specialities.com/natural-specialities/en/8463-products-list.html) supplied the following tannic acids (CAS: 1401-55-4): non-purified Chinese gallnuts powder and highly purified Sumac powder for use in a 50:50 mixture as the hydrolysable tannic acid source for p1, c1, p2, and c2. Fibrecrafts (http://www.georgeweil.com/Default.aspx) and Verfmolen “de Kat” (http://www.verfmolendekat.com/en/home.html) supplied the gall nuts powder for use in p3 and c3. Phoenix Calico Limited supplied plain woven, scoured, bleached 100% cotton fabric (135 g/m^2^). Parkin Fabrics Limited (http://www.parkinfabrics.co.uk/) supplied undyed, unstiffened, natural pinokpok (abaca). Whaleys (Bradford) Limited (http://www.whaleys-bradford.ltd.uk/index.htm) supplied 100% wool botany serge (190 g/m^2^). Professor J. Shao, Zhejiang Sci-Tech University, Hangzhou, PRC, kindly supplied the scoured, degummed plain weave silk (220 g/m^2^).

| 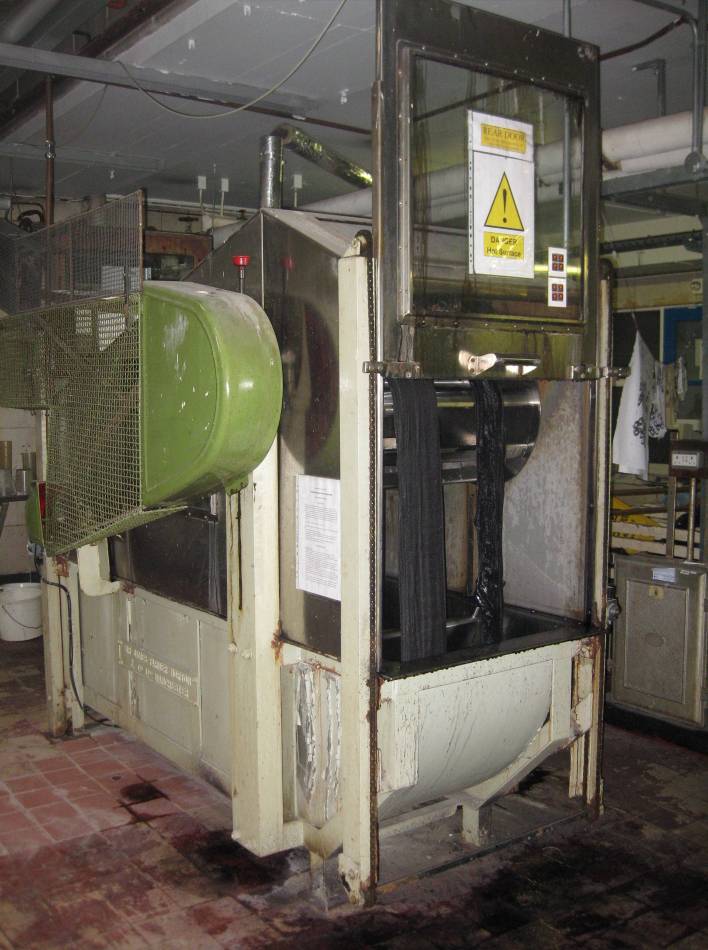  Figure 1. Winch dyeing of wool and silk textiles | 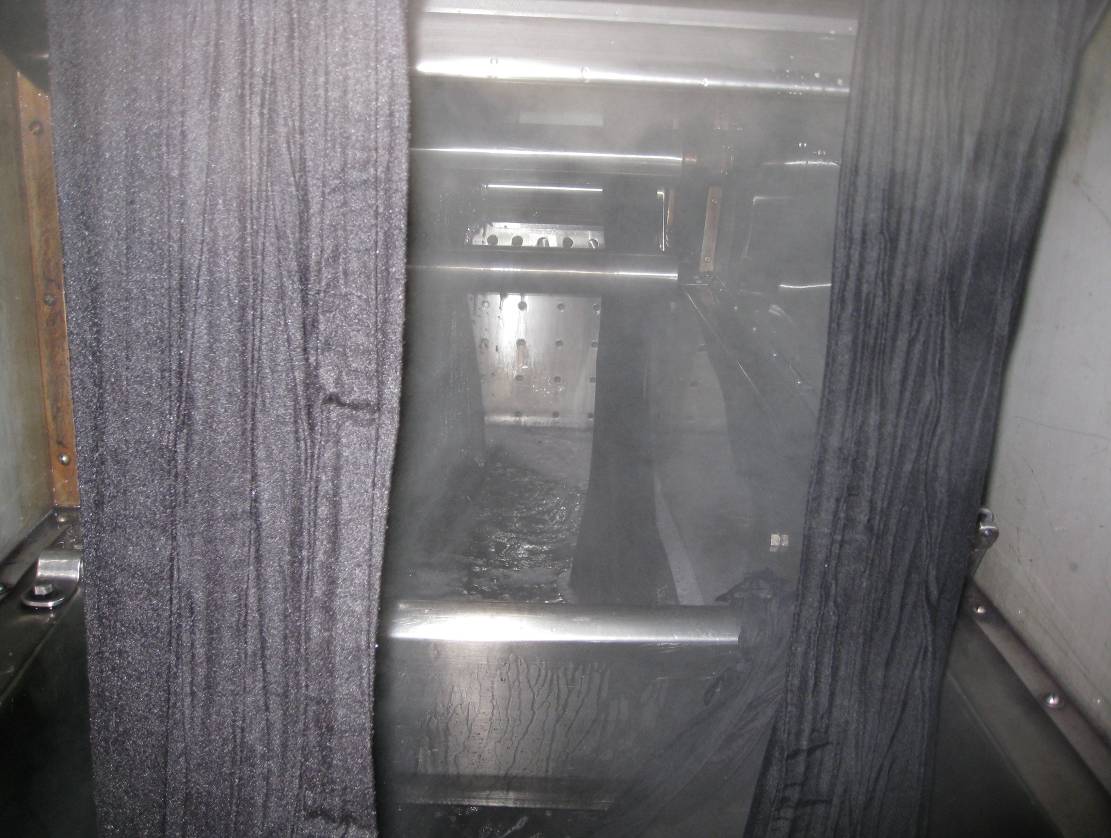  Figure 2. Ropes of wool and silk are moved through the dye solutions in the Winch machine |
| --- | --- |
| 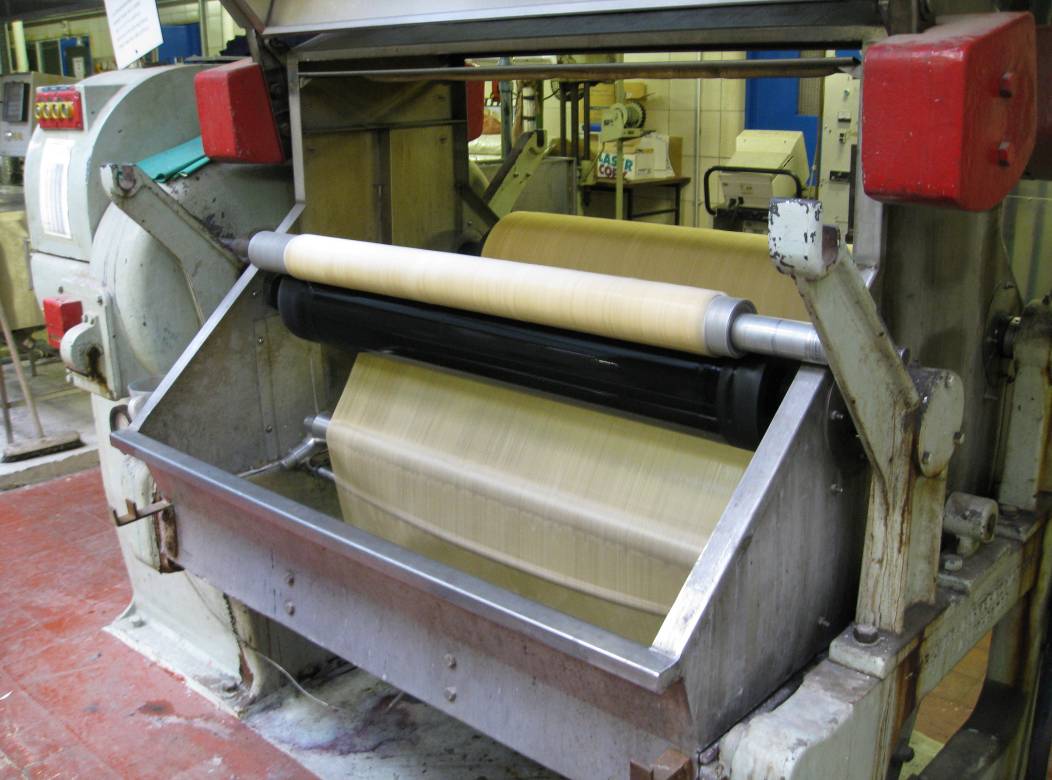  Figure 3. Abaca and cotton textiles being washed on a Jigger machine | 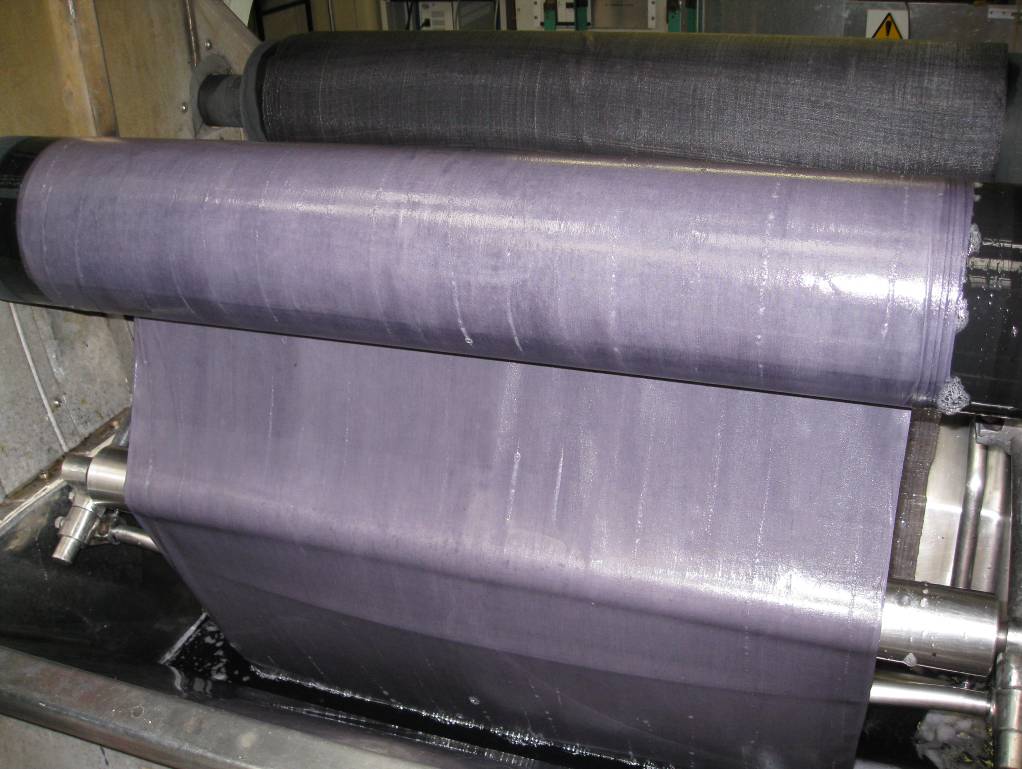  Figure 4. Cotton (front) and abaca (back) textiles move between rollers and through the dye solution in the Jigger machine |
