# Supplementary material for: Production and validation of model iron-tannate dyed textiles for use as historic textile substitutes in stabilisation treatment studies
Source: Chem Cent J. 2012 May 22;6:44. doi: 10.1186/1752-153X-6-44 (PMC3495704; doi:10.1186/1752-153X-6-44)
Supplement: Additional file 3 — Experimental section. Text only [37,38,67]. [file 1752-153X-6-44-S3.doc]

Additional file 3: Experimental Section

**1 Dyeings**

The dyeings were performed on a Winch and a Jigger machine at the University of Manchester’s dyehouse. Further information including material sources can be found in Additional file 2.

**2 Analysis**

Analyses of unaged substitute textiles were undertaken within 28 months of substitute textile production. For 12 months or less the textiles were stored in partially lit, uncontrolled environmental conditions, after which they were stored in dark, uncontrolled environmental conditions. It is assumed that any changes in substitute textile properties as a result of storage during this time are negligible.

The substitute textiles were prepared into sheets of approximately 30 cm by 45 cm before analysis. Smaller pieces of U, p1 and c1 substitute textiles (at least 10 cm by 15 cm) were stacked in two stacks (one for dyed and one for undyed samples) and subjected to accelerated ageing at 80°C and 58% RH for 1, 2, 3, and 4 weeks in a Sanyo Gallenkamp Environmental Chamber. The stacks were arranged in the order of abaca, cotton, silk, and wool from the shelf upwards. The stacks were not rotated during ageing but were moved around on the shelf throughout ageing to counter any location-dependant variations in temperature and relative humidity in the chamber. Accelerated ageing is frequently used in conservation science to accelerate the degradation of materials for Arrhenius tests, and simulation or comparison studies [67]. The greater the accelerated ageing conditions vary from ambient conditions, the faster the degradation but the greater the risk that the degradation is not identical to that occurring naturally. The environmental conditions used in this study are similar to those used in iron gall ink studies (80°C, 65% RH) [37].

2.1 XRF

A Bruker ArtTax µ-XRF spectrometer with a molybdenum X-ray tube was used for semi-quantitative analysis of the elemental content of the substitute textiles. Single thicknesses of substitute textiles were analysed on filter paper for 100 s, using a 1.5 mm collimator, 50 kV, and 500 µA in air. 8 analyses from different areas per substitute textile were obtained with the same textile alignment. The net peak areas of elements (A_e_) and the Compton peak (A_c_) were calculated by ArtTax4.9 software using the Bayes deconvolution method. The net elemental peak areas were normalised to the Compton peak using the following equation:

$$Ratio=\left(\frac{A_{e}}{A_{c}}\right)\times1000$$

The mean ratios, and standard deviation of iron and copper were calculated from the 8 ratios calculated per substitute textile. Background analyses of the support beneath the textile samples indicated background ratios (normalised to the Compton peak) of iron and copper to be no more than 30 and 10, respectively.

Analysis of material from museum objects occurred using the same experimental conditions as above with small samples such as a few short strands being adhered to a carbon tab on filter paper for analysis.

2.2 SEM-EDX of substitute textile cross-sections

Dyed (p1 and c1) and undyed substitute textile fabrics were mounted in resin (bisphenol A-epichlorohydrin) and prepared as polished cross-sections.

Analysis of the cross-sections was undertaken using an Hitachi S-4800 Field Emission SEM and an Hitachi variable pressure S-3700N SEM (operating at 30Pa). The SEMs were operated at 20 kV and a 12 mm working distance for all analyses. Analysis was conducted using Oxford Instruments energy dispersive X-ray analysers with INCA software. EDX spectra were collected for varying livetimes after optimisation of the iron peak versus total time taken for analysis: 200 s for abaca and silk; 200-300 s for cotton and 500-1000 s for wool. Dyed and undyed samples of the same material were analysed using the same conditions for comparison.

2.3 Surface pH analysis

Individual sheets of substitute textile were laid on a clean glass sheet and a drop of deionised water added. It was ensured that the water dispersed into the fabric rather than remaining as a drop on the surface prior to undertaking the analysis. In some cases more than one drop of water was needed to wet the textile sufficiently for successful measurement. A Mettler Toledo InLab®Surface pH electrode attached to a Hanna Instruments HI2210 pH meter with temperature probe was then applied to the wetted area and held in place until the pH value stabilised. Ten analyses per substitute textile were made on randomly selected locations of randomly selected textile sheets. pH 4.01 and pH 7.01 buffer solutions were used to calibrate the equipment prior to analysis.

Samples of museum objects were analysed as above one and four times depending on sample size.

2.4 Tensile testing

70 – 100 mm long strips of cotton and silk textiles (10 mm wide) and strips of abaca textiles (11 fibre bundles wide) were tested using an Instron 4411 tensile tester with 500 N static load cell and Series IX software. The warp direction of the cotton, abaca and silk fabrics was tested. The strips had been conditioned to approximately 21°C and 50% RH overnight before testing. Between eight and ten strips were analysed per sample (as sample size allowed) using a 50 mm gauge length and 10 mm min^-1^ extension speed as used by Garside, Wyeth and Zhang [38]. The samples were placed centrally, vertically, and slightly slack in the clamps. Wool was not tested because its high tensile strength caused excessive slippage of the strips from the jaws when extended under these conditions. Yarn testing would be more suitable but was not possible in the timeframe of the project.

Exponential trend lines were fitted to tensile testing data using MS Excel.

2.5 Spectrophotometry

Spectrophotometric data was collected using a Konica/Minolta CM-2600d spectrophotometer, Spectramagic 3.60 software and the following settings: SCI+SCE, medium aperture, UV included, 10° observer and D65 illuminant. The spectrophotometer was calibrated using a white standard before analysis and the textiles were analysed on black velvet.

2.5.1 Unaged textiles:

10 randomly selected sheets of each model textile were analysed in 3 randomly selected locations to provide mean, standard deviation, and coefficient of variation L*, a*, b* values of SCI/100 data (Table 5). The standard deviation for SCE/100 data was not collected.

2.5.2 Aged textiles:

Average L*, a*, b* values were calculated by the software from 5 randomly selected locations on each aged model textile. CIE2000 was used to calculate the ΔE_00_*, ΔL*, Δa*, Δb* from SCE/100 data from the aged textile compared to the unaged equivalent textile. SCE/100 data was chosen for evaluation of colour changes in aged model textiles because it excluded the specular component which could be a source of error in the textiles with more reflective surfaces such as abaca and silk. However, SCI and SCE data for the unaged model textiles differed little from each other; typically less than 1.2% variation in L*, and less than 10% variation in a* and b*. Only U, p1, and c1 textiles were included in this study.

2.5.3 Museum objects

The small aperture rather than medium aperture was used to analyse up to three areas of the sample as sample size allowed. All other conditions were the same as for spectrophotometry of model textiles.
